# Comprehensive evaluation of the nutritional value and contaminants of alfalfa (*Medicago sativa* L.) in China

**DOI:** 10.3389/fnut.2025.1539462

**Published:** 2025-02-10

**Authors:** Xiaoyan Zhu, Wenxue Chen, Mengyao Li, Boshuai Liu, Shumin Zhao, Menglin Hu, Jiajing Li, Defeng Li, Yinghua Shi, Hao Sun, Chengzhang Wang

**Affiliations:** ^1^Department of Animal Nutrition and Feed Science, College of Animal Science and Technology, Henan Agricultural University, Zhengzhou, China; ^2^Henan Key Laboratory of Innovation and Utilization of Grassland Resources, Zhengzhou, China; ^3^Henan Herbage Engineering Technology Research Center, Zhengzhou, China

**Keywords:** alfalfa, mycotoxin, microorganism, heavy metal, food safety evaluation, Nemerow contamination index method

## Abstract

**Introduction:**

Alfalfa is used as a feed source for animals and plays an important role in animal nutrition. Nutritional value analysis and contamination evaluation are essential for sustainable utilisation to ensure the quality and safety of alfalfa.

**Aim:**

We aimed to evaluate the nutritional value and safety of alfalfa in five major regions in China—the Northeast Plain (NP), Inner Mongolia Plateau (IMP), Northwest Desert Oasis (NO), Loess Plateau (LP), and Huanghuaihai Plain (HP).

**Methods:**

Ninety-five samples representing 44 large-scale pratacultural companies were collected and analysed.

**Results:**

The average nutritional values of alfalfa hay were 16.43% crude protein (CP), 44.01% neutral detergent fibre (NDF), 33.22% acid detergent fibre (ADF), 10.78% crude ash (Ash), and 91.90% dry matter (DM), which were better in the NO and IMP areas; in particular, the α-linolenic acid value of alfalfa was the highest in the IMP area (p < 0.05). The detection rates of mycotoxins, microorganisms, and heavy metals were 100%. The over-standard rates of total bacteria count and total mould count were 66.7% and 75.8%, respectively, while the over-standard rate of chromium in heavy metals was as high as 87.9%.

**Discussion:**

This study establishes the nutrient composition and harmful component profiles of alfalfa hay in five major planting areas for the first time, which provides a valuable reference for the safe and sustainable utilisation of alfalfa in livestock feed.

## 1 Introduction

Many forage lands have been established to increase pasture yield to meet the ever-increasing demand for food (1). Legumes are generally used for grassland planting. Alfalfa (*Medicago sativa* L.), one of the most widely grown forage crops worldwide, is a high-yielding perennial forage that can rapidly regenerate many new shoots after harvest and can be harvested multiple times during the growing season every year (2, 3). It has high nutritional content (4, 5), high digestibility, and unique proportions of structural and non-structural components (6), which can not only be used as a feed source for animals, but also as a green leafy vegetable for human consumption (7). In addition, the crude protein (CP) content of alfalfa is approximately 15–20%, which makes it one of the highest protein-producing plants per hectare (8). From 2015 to 2017, “Central Document No. 1” pointed out that it is necessary to speed up the development of grass and animal husbandry, which vigorously supports the cultivation of forage and other forage materials and accelerates the construction of a modern forage industry system. The introduction of these series of policies has promoted the development of the forage industry, reflected the importance and urgency of forage industry, and also ensured excellent quality. However, owing to the late start of China’s alfalfa industry, alfalfa production companies and grass and animal combination enterprises rarely measure the nutritional and safety components before using alfalfa products. Most product transactions are based on sensory standards for quality evaluation, and the quality of domestically produced alfalfa hay is not effectively supervised and guaranteed (9). Nutritional value analysis and contamination evaluation are essential for sustainable utilisation to ensure the quality and safety of alfalfa hay.

Many studies have shown that there are great differences in the nutrient composition of alfalfa in different producing areas, and the origin of alfalfa should be one of the main bases for selecting alfalfa products (10, 11). Wang et al. (12) detected mycotoxins in feed and feed stocks, and identified that alfalfa samples were severely contaminated with aflatoxin B1 (ABF1, 168 μg kg^−1^), deoxynivalenol (DON, 227 μg kg^−1^), and zearalenone (ZEN, 1631 μg kg^−1^). Zhang et al. (13) compared the absorption and transportation abilities of heavy metals in four alfalfa varieties and suggested that alfalfa was susceptible to heavy metal accumulation and not suitable as a sole source of feed stock. The above studies have shown that there are differences in the nutritional values of alfalfa of different origins and that they are susceptible to contamination by mycotoxins and heavy metals. Currently, there is a lack of comprehensive evaluation of the nutrients [crude protein (CP), neutral detergent fibre (NDF), acid detergent fibre (ADF), ether extract (EE), crude ash (Ash), mineral elements, amino acids, and fatty acids] and contaminants (mycotoxins, microorganisms, heavy metals) in alfalfa hay. The quality distribution and contamination of alfalfa products are unclear. Only by understanding the actual nutritional values of alfalfa hay and combining it with the characteristics and hazard degree of contaminants can we take corresponding preventive and control measures and provide a scientific basis to the enterprises for safer production.

**Figure 1:**
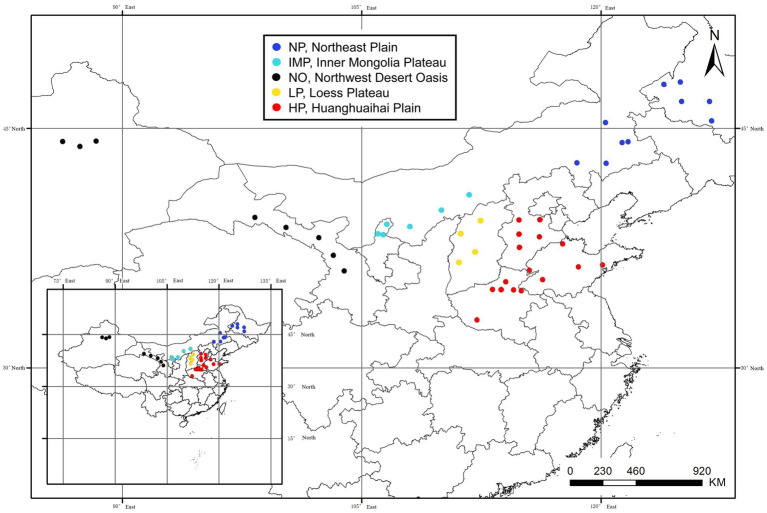
Distribution of 44 enterprises among five major planting areas. The five areas include the Northeast Plain (NP), Inner Mongolia Plateau (IMP), Northwest Desert Oasis (NO), Loess Plateau (LP), and Huanghuaihai Plain (HP).

In this study, we aimed to establish the nutrient composition and harmful component profiles of alfalfa hay from 44 enterprises in five major planting areas in China (Figure 1) and provide a valuable reference for the safe and sustainable utilisation of alfalfa in human food and livestock feed.

## 2 Materials and methods

### 2.1 Alfalfa sample and sampling procedure

Alfalfa hay samples were collected in summer, and more than 90% of alfalfa samples were produced between the 30° and 45° northern latitude range. The main alfalfa production regions are divided into five natural regions (Figure 1; Table 1), which include the Northeast Plain (NP), Inner Mongolia Plateau (IMP), Northwest Desert Oasis (NO), Loess Plateau (LP), and Huanghuaihai Plain (HP) (14–16). A total of 95 samples were collected, representing 44 large-scale pratacultural companies from the five natural regions. The alfalfa hay samples were widely distributed, which including different varieties, cutting times, growth periods, grades, storage methods, and production processes, and they were treated with uniform pre-treatment and chemical analysis methods to eliminate systematic errors.

**Table 1:** Description of the five sampling regions.

Sampling sites^a^	Latitude	Longitude	Category	Altitude (m)	Mean temperature in summer (°C)	Annual precipitation (mm)	Summer precipitation (mm)
NP	43°34′ to 46°39′N	120°25′ to 125°53E	Humid/sub-humid mid-temperate monsoon climate	120–300	14.25–26.00	382.75	299.25
IMP	38°33′ to 40°25′N	106°3′ to 109°43′E	Arid temperate continental climate	1,000–1700	13.00–26.50	263.50	171.50
NO	38°14′ to 44°20′N	86°25′ to 102°31′E	Arid moderate /warm temperate continental climate	680–1,480	12.75–26.50	188.75	92.00
LP	39°12′ to 39°19′N	112°29′ to 112°42′E	Warm temperate monsoon climate	800–1,300	15.00–28.00	516.00	308.00
HP	32°58′ to 38°18′ N	112°12′ to 120°33′ E	Humid/sub-humid warm temperate monsoon climate	34–120	18.75–28.50	706.00	350.70

a NP, Northeast Plain; IMP, Inner Mongolia Plateau; NO, Northwest Desert Oasis; LP, Loess Plateau; HP, Huanghuaihai Plain.

The products of the same specification produced in the same storage yard of the same production unit are regarded as the same inspection batch; 20 bundles of representative hay bales were randomly selected from the same inspection batch, and each bale was sampled with alfalfa hay sampler (12 mm inside diameter and 300 mm long). The sampler probe and cross section of alfalfa bale was maintained at 90 degrees within 15 cm of the bale edge to avoid sampling on the alfalfa bale surface. The samples were pooled so that each pooled sample contained 20 small probe samples (17) and then sealed in a polyethylene plastic bag and sent to Henan Key Laboratory of Innovation and Utilization of Grassland Resources immediately. Each sample was divided into two parts. One portion was dried in a forced-air oven at 65°C for 48 h and then crushed using a grinder with a 1 mm sieve to determine the nutritional ingredients and heavy metals. The non-dried portion was processed immediately for microbial and mycotoxin characterisation, as described below.

### 2.2 Determination of conventional nutrients

The alfalfa hay contents of dry matter (DM), CP, Ash, and EE were measured by methods No. 934.01, 990.03, 942.05 and 920.39 of Association of Official Analytical Chemists (18) (AOAC, 1990). CP (N × 6.25) was analysed according to the Kjeldahl method (Foss, Kjeltec 8,400). NDF and ADF were examined using an Ankom Fibre Analyzer (Ankom Technology, Fairport, NY, United States) according to the method described by Van Soest et al. (19).

### 2.3 Determination of amino acids and fatty acids

The amino acid (AA) and fatty acid (FA) composition of alfalfa hay samples were measured according to the national standard of the People’s Republic of China (20, 21). The samples were hydrolysed with 6 N HCl at 110°C for 22 h and then quantitative and qualitative analyses of 16 AA were performed using ion exchange chromatography with an automatic AA analyser (Hitachi L-8800, Tokyo, Japan). Tryptophan was analysed using HPLC (Agilent 1,200 Series, Santa Clara, CA, United States) according to the method described by Xu et al. (22). FA composition was determined using gas chromatography coupled with a flame ionisation detector (GC-FID; Shimadzu GC-2010 Plus, Japan) and a 100% cyanopropyl polysiloxane capillary column (SP 2560, 100 m, 0.25 mM i.d. and 0.20 μM film thickness, Sigma-Aldrich, St. Louis, MO). The gas flows for the carrier gas (N_2_), make-up gas, H_2_, and synthetic air were 1.0, 30, 40, and 400 mL min^−1^, respectively. The injector and detector temperatures were maintained at 270°C and 280°C, respectively. The initial oven temperature of 100°C was held for 13 min, increased at 10°C min^−1^ to 180°C, held for 6 min, increased at 1°C min^−1^ to 200°C, held for 20 min, and then increased at 4°C min^−1^ to 230°C and maintained for 10.5 min. Nitrogen was used as the carrier gas and 1.0 μL of sample was injected; the split ratio was 100:1. The concentration of each FA was expressed as a percentage of the total FA identified in the alfalfa hay samples. The composition of saturated fatty acids (SFA), monounsaturated fatty acids (MUFAs), and polyunsaturated fatty acids (PUFAs) were obtained from individual FA percentages.

### 2.4 Mineral element and heavy metal determination

Weigh 2 g of alfalfa hay samples in a triangular flask and add 20 mL mixed acid (nitric acid: perchloric acid is 4:1, Analytical Reagent Grade) to soak overnight. Heat the samples at 150°C for 3 h, and adjust the temperature to 220°C after the smoke becomes shallow, leaving 5 mL of the solution. Cool and add deionized water to drive off the acid, and continue to digest at 150°C for about 5 h until the solution is reduced to 2–3 mL. Transfer the solution to a 25 mL volumetric flask, dilute to volume with deionized water, mix and filter, and then analyze the contents of mineral elements and heavy metals in the samples (23). The contents of calcium (Ca), phosphorus (P), potassium (K), magnesium (Mg), zinc (Zn), and copper (Cu) in alfalfa hay were measured using an inductively coupled plasma-mass spectrometer (Agilent7800, United States). Cadmium (Cd), chromium (Cr), and lead (Pb) contents in alfalfa hay were analysed using an atomic absorption spectrophotometer (Hitachi Z2000, Japan). The arsenic (As) content was determined using an atomic fluorescence spectrophotometer (Persee PF6, China).

### 2.5 Microbe determination

Alfalfa hay sample (10 g) was mixed with 90 mL sterile potassium phosphate buffer (pH = 7) and agitated for 30 min at 260 rpm in a Stomacher 400 Laboratory Blender (Seward, London, United Kingdom). The liquid phase was subsequently serially diluted (102 to 107) with the same potassium phosphate buffer, and 100 μL aliquots of each dilution were spread in triplicate onto nutrient agar (NA; Difco, Aobox, China) for the enumeration of total bacterial count (TBC) and onto rose Bengal medium (RBM; Difco) for the enumeration of total mould count (TMC). The NA plates were incubated at 30°C for at least 48 h, while the RBM plates were incubated at 25°C for 72 h. Only plates with colony numbers in the range of 30–300 were counted.

### 2.6 Mycotoxin determination

The quantitative identification of aflatoxin B1 (AFB1), deoxynivalenol (DON), and zearalenone (ZEA) in alfalfa hay samples was performed using enzyme-linked immunosorbent assay using test kits [Reorder#: COKAQ8000 (96 wells), COKAQ4000 (96 wells), COKAQ5100 (96 wells), manufactured in Singapore, licensed by Romer Labs^®^, Lnc. U.S.A.], and followed the manufacturer’s instructions.

### 2.7 Grey relational analysis method for evaluating nutritional value

Nutritional evaluation of alfalfa hay was performed using the grey relational analysis (GRA) method using the following formula (24):

For comprehensive evaluation indices $k\;\left(1\leq k\leq n\right)$ and comprehensive alternatives $i\;\left(1\leq i\leq m\right)$ , a sample matrix $\left\{X_{i}\left(k\right)\right\}$ was constructed. These indices usually have different dimensions and magnitudes. Therefore, the normalising procedure may be performed using the mean.

According to the GRA theory, $\left\{X_{0}\left(k\right)\right\}$ was set as the reference sequence. The series is a comparison. Using the GRA, we can obtain the coefficient number for the *k*th index of the *it*h alternative. The matrix can be written as:


(1) $$\Delta_{i}\left(k\right)=\left|X_{0}\left(k\right)-X_{i}\left(k\right)\right|$$

Then, the grey relational coefficient matrix is obtained, with the elements formulated as:


(2) $$\begin{array}{l} \varepsilon_{i}\left(k\right)=\\ \frac{\min\left(i\right)\min\left(k\right)\left|X_{0}\left(k\right)-X_{i}\left(k\right)\right|+\rho \max\left(i\right)\max\left(k\right)\left|X_{0}\left(k\right)-X_{i}\left(k\right)\right|}{\Delta_{i}\left(k\right)+\rho \max\left(i\right)\max\left(k\right)\left|X_{0}\left(k\right)-X_{i}\left(k\right)\right|} \end{array}$$

where $\left|X_{0}\left(k\right)-X_{i}\left(k\right)\right|$ is the absolute difference between the $X_{0}$ and $X_{i}$ sequences at the k th point. $\varepsilon_{i}\left(k\right)$ is the grey relational coefficient of the k th index of the i th alternative. $\min\left(i\right)\min\left(k\right)|X_{0}\left(k\right)-X_{i}\left(k\right)|$ is the secondary minimum and $\max\left(i\right)\max\left(k\right)|X_{0}\left(k\right)-X_{i}\left(k\right)|$ is the secondary maximum. The factoris $\rho \in \left[0,\cdot 1\right]$ the distinguishing coefficient and is usually set to 0.5.

Based on the methodology of GRA and the index weights $\omega \left(k\right)$ , the final calculation model can be deduced as:


(3) $$\gamma_{i}=\overset{n}{\underset{k=1}{\sum }}\omega \left(k\right)\varepsilon_{i}\left(k\right)$$

where $\gamma_{i}$ is the result of the evaluated alternatives, $\varepsilon_{i}\left(k\right)$ is the grey relational coefficient matrix of indices, and $\omega \left(k\right)$ is the weight of the evaluated indices. According to the principle of maximum correlation, the evaluation alternative can be sorted; the larger the, the $\gamma_{i}$ better the alternative.

### 2.8 Contamination assessment of alfalfa hay

To evaluate the contamination of the alfalfa hay samples, the single factor contamination index method and the Nemerow contamination index method were used with the following formulas (25, 26):


(4) $$P_{i}=\frac{c_{i}}{s_{i}}$$

(5) $$I=\sqrt{\frac{\left(P_{i}^{2}\max+P_{i}^{2}ave\right)}{2}}$$

where $P_{i}$ is the over-limit ratio of safety component of alfalfa hay, i is the species of safety component, $C_{i}$ is the tested single safety component concentration of alfalfa hay, and $S_{i}$ is the limited standard of safety component of alfalfa hay (Supplementary Table S1); I is the quality of various types of comprehensive contamination indices, $P_{i}\max$ represents the maximum value of one safety component, $P_{i}ave$ is the average value. Meanwhile, the contamination hierarchy standard was used to classify alfalfa hay (Supplementary Table S2).

### 2.9 Statistical analyses

Data pre-processing was performed using Excel 2016. General linear model analysis and one-way analysis of variance were conducted using SPSS 20.0. The results were expressed as “mean ± SD.” Differences among means in different treatments were tested using Duncan’s multiple range tests. Significant differences were determined at 0.05 levels.

## 3 Results

### 3.1 Conventional nutrients of alfalfa hay in different regions

**Figure 2:**
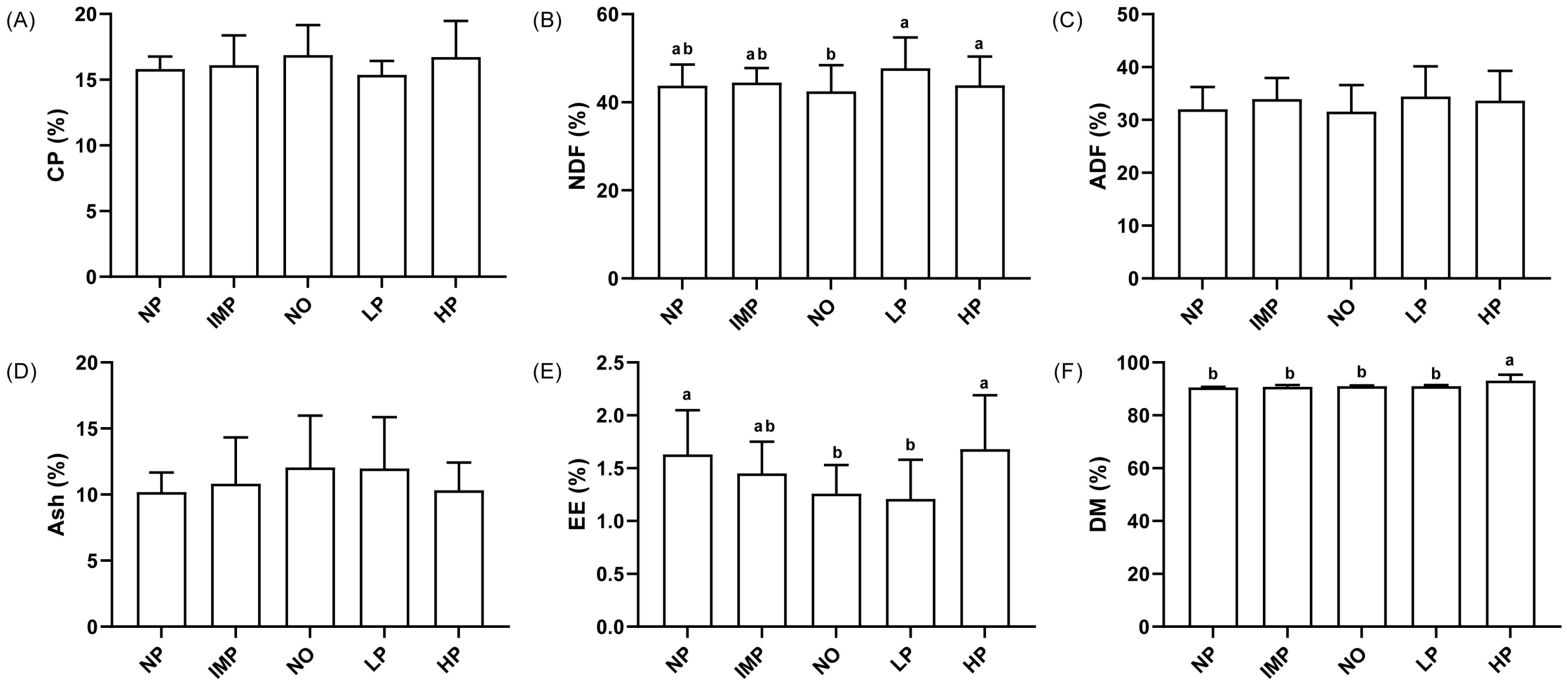
Conventional nutrients contents of alfalfa hay (%). Conventional nutrients include crude protein (CP), neutral detergent fibre (NDF), acid detergent fibre (ADF), Ash, ether extract (EE), dry matter (DM) in five regions **(A–F)**. The five areas include the Northeast Plain (NP), Inner Mongolia Plateau (IMP), Northwest Desert Oasis (NO), Loess Plateau (LP), and Huanghuaihai Plain (HP). Different lower letters indicate that the differences are significant at 0.05 levels.

The results of conventional nutrient contents of alfalfa hay in different regions are shown in Figures 2A–F. There were no differences in CP, ADF, and Ash contents in alfalfa hay samples among the five regions (*p* > 0.05), whereas NDF, EE, and DM contents showed significant differences (*p* < 0.05). The NDF content in the LP region was the highest, and it was especially higher than that in the NO region (*p* < 0.05). The EE content was similar between the NP and HP regions (*p* > 0.05) and the NO and LP regions (*p* > 0.05). The NP and HP regions showed higher EE content than the NO and LP regions (*p* < 0.05). The DM content was the highest in the HP region (*p* < 0.05). The average contents of conventional nutrients in alfalfa hay in northern China were as follows: CP, 16.43%; NDF, 44.00%; ADF, 33.22%; Ash, 10.78%; EE, 1.53%; and DM, 91.90%.

### 3.2 Mineral elements of alfalfa hay in different regions

**Figure 3:**
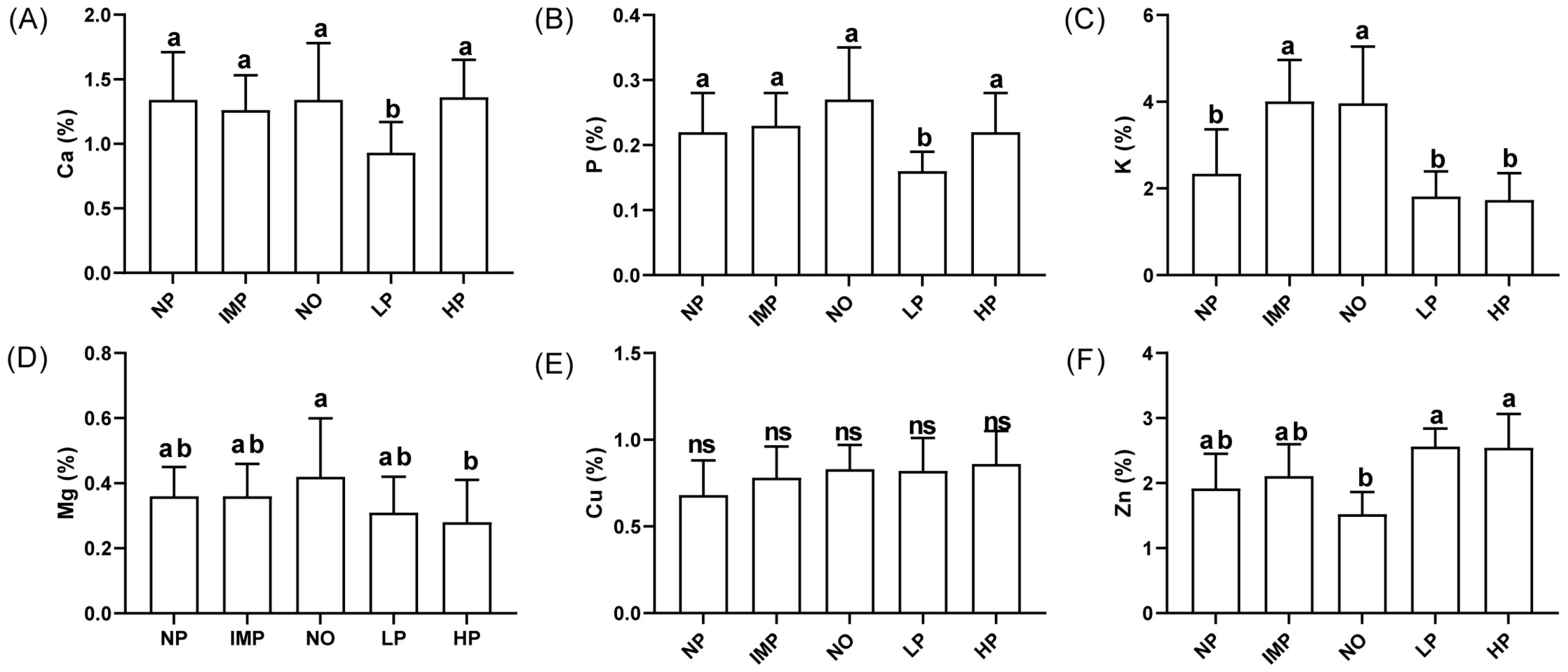
Mineral elements contents of alfalfa hay (%). Mineral elements include Ca, P, K, Mg, Cu, Zn in five regions **(A–F)**. The five areas include the Northeast Plain (NP), Inner Mongolia Plateau (IMP), Northwest Desert Oasis (NO), Loess Plateau (LP), and Huanghuaihai Plain (HP). Different lower letters indicate that the differences are significant at 0.05 levels.

The Ca, P, K, Mg, Cu, and Zn contents of alfalfa hay in different regions are shown in Figures 3A–F. The Cu content of alfalfa hay in the five regions was similar (*p* > 0.05). Alfalfa hay samples from the NO region showed higher Ca, P, K, and Mg contents and lower Zn content, while the LP region showed lower Ca, P, K, and Mg contents and higher Zn content. Ca and P contents in the LP region were significantly lower than those in the other four regions (*p* < 0.05). K content was higher in the IMP and NO regions than in the NP, LP, and HP regions (*p* < 0.05). Mg content in the NO region was significantly higher than that in the HP region (*p* < 0.05), and Zn content in the LP and HP regions was significantly higher than that in the NO region (*p* < 0.05).

### 3.3 Fatty acids of alfalfa hay in different regions

**Figure 4:**
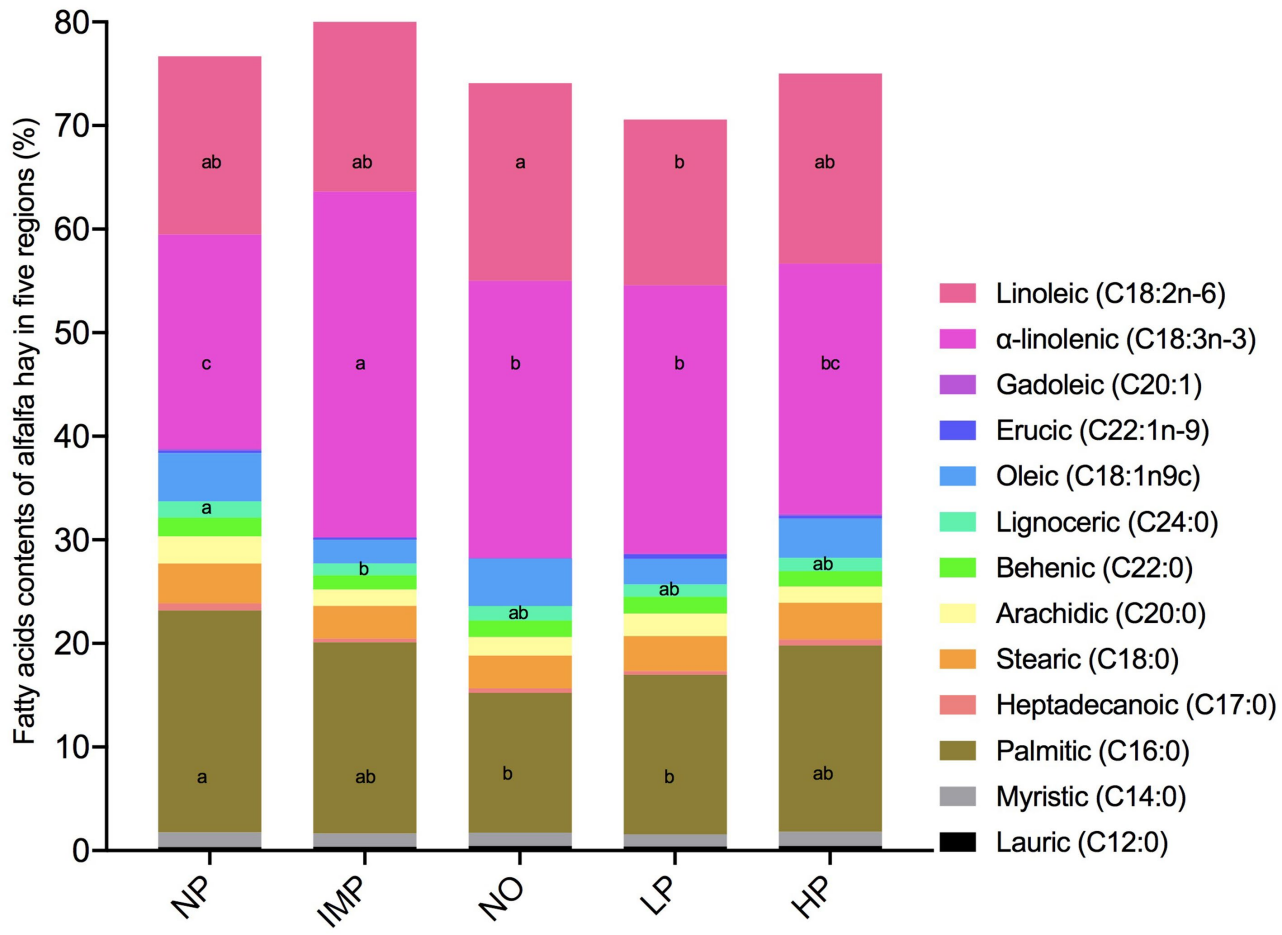
Fatty acids contents of alfalfa hay in the five areas (%). The five areas include the Northeast Plain (NP), Inner Mongolia Plateau (IMP), Northwest Desert Oasis (NO), Loess Plateau (LP), and Huanghuaihai Plain (HP). Saturated fatty acids (SFA) include Lauric (C12:0), Myristic (C14:0), Palmitic (C16:0), Heptadecanoic (C17:0), Stearic (C18:0), Arachidic (C20:0), Behenic (C22:0) and Lignoceric (C24:0). Monounsaturated fatty acids (MUFA) include Oleic (C18:1n9c), Erucic (C22:1n-9) and Gadoleic (C20:1). Polyunsaturated fatty acids (PUFA) include n-6 PUFA and n-3 PUFA, which is Linoleic (C18:2n-6) and *α*-linolenic (C18:3n-3), respectively. “Means ± SD” within rows with different superscript letters differ (*p* < 0.05).

Thirteen types of FA in alfalfa hay from five regions were determined, including eight different SFAs, three different MUFAs, and two different PUFAs (Figure 4). Except for lauric acid, the other seven different saturated FA were the highest in the NP region, resulting in an increase in total SFA in the NP region (*p* < 0.05). The MUFA of alfalfa hay in the NP region was the highest, but there was no significant difference among the other regions (*p* > 0.05). Alfalfa hay in the IMP region had the highest PUFA (*p* < 0.05), mainly because of the greater *α*-linolenic acid content than the other four regions (*p* < 0.05), whereas the PUFA and α-linolenic acid contents in the NP region were the lowest (*p* < 0.05). Moreover, the linoleic acid content in the NO region was higher than that in the LP region (*p* < 0.05).

### 3.4 AAs of alfalfa hay in different regions

**Figure 5:**
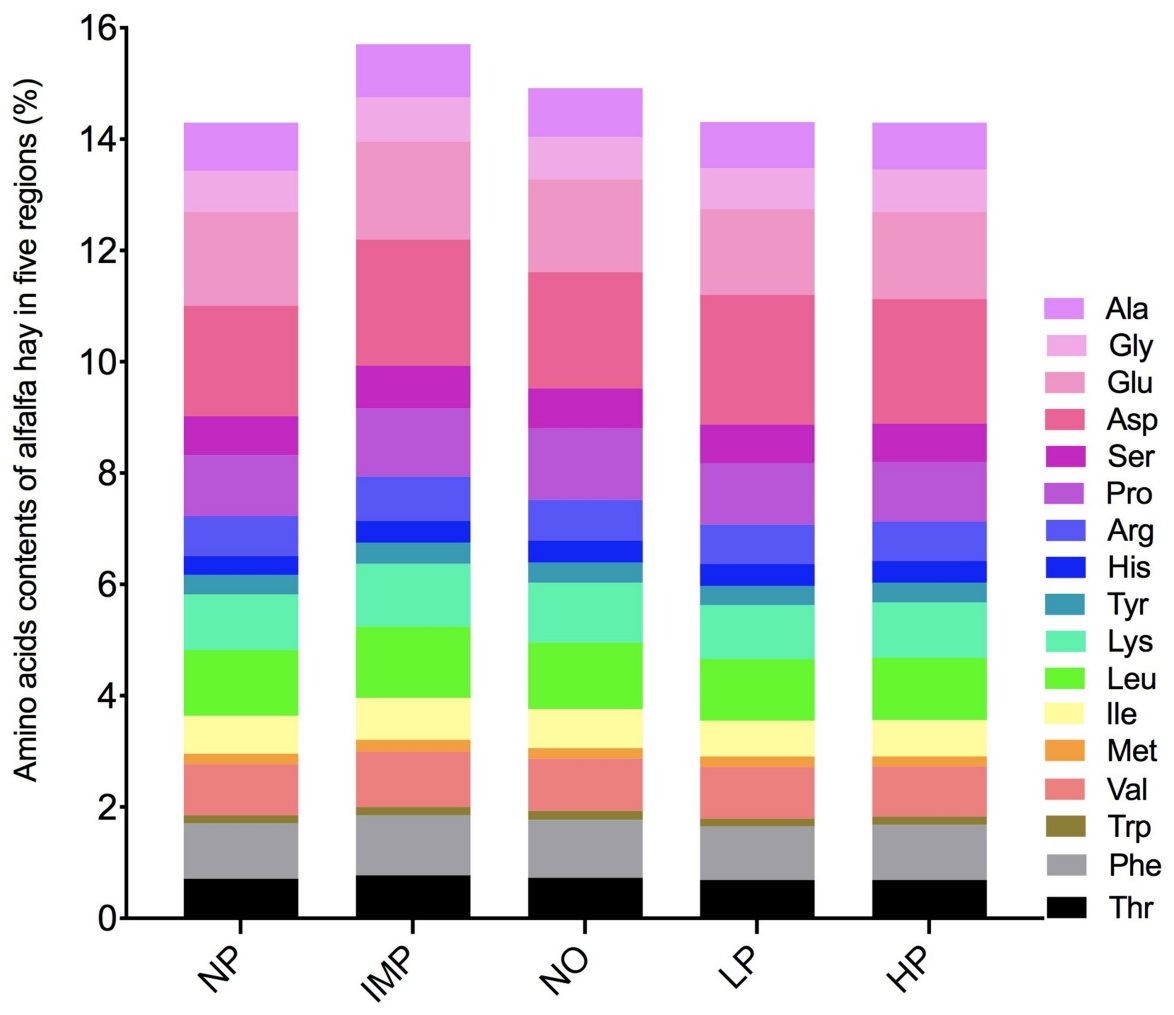
Amino acids contents of alfalfa hay in the five areas (%). The five areas include the Northeast Plain (NP), Inner Mongolia Plateau (IMP), Northwest Desert Oasis (NO), Loess Plateau (LP), and Huanghuaihai Plain (HP). Amino acids (AA) include Threonine (Thr), Phenylalanine (Phe), Tryptophan (Trp), Valine (Val), Methionine (Met), Isoleucine (Ile), Leucine (Leu), Lysine (Lys), Tyrosine (Tyr), Histidine (His), Arginine (Arg), Proline (Pro), Serine (Ser), Aspartic acid (Asp), Glutamic acid (Glu), Glycine (Gly) and Alanine (Ala). Essential amino acid (EAA) includes Thr, Val, Met, Ile, Leu, Phe, Lys, and Try.

The AA contents of alfalfa hay in different regions are shown in Figure 5. There was no significant difference in 17 types of AAs among the five regions (*p* > 0.05), indicating that the AA composition and contents in alfalfa hay did not change with regional changes.

### 3.5 Grey correlation analysis on alfalfa hay nutrients

In this study, seven important nutritional indices, namely, CP, NDF, ADF, Ca, P, EAA, and *α*-linolenic, were used as a basis to determine the nutritional values. The reference sequence was determined according to the optimal values of the seven nutritional indices of alfalfa hay in each region. The optimal values were CP: 16.88%, NDF: 42.49%, ADF: 31.58%, Ca: 1.36%, P: 0.27%, EAA: 6.35%, and α-linolenic: 33.40%. Dimensionless data (Supplementary Table S3), absolute differences between *X_0_* and *X_1_* (Supplementary Table S4), and correlative coefficients (Supplementary Table S5) of each nutritional composition are determined by formula calculation (Equations 1, 2). The expert scoring method was used to assign different weights to each nutritional index, and the weight coefficients of each nutritional index were as follows: *ω*_CP_ = 0.3, *ω*_NDF_ = 0.2, *ω*_ADF_ = 0.2, *ω*_Ca_ = 0.05, *ω*_P_ = 0.05, *ω*_EAA_ = 0.1, and *ω*_α-linolenic_ = 0.1. Substituting the weight coefficient into the weighted correlation formula (Equation 3), the weighted correlation degrees of the nutritional value of alfalfa hay in different regions were in the order of (ranking from highest to lowest) (Table 2) NO (0.9067) > IMP (0.8885) > NP (0.8177) > HP (0.7744) > LP (0.7147).

**Table 2:** Correlation degrees of nutritional value of alfalfa bale in five regions.

Item^a^	$\gamma_{i}$ ^b^	Weighted order
NP	0.8177	3
IMP	0.8885	2
NO	0.9067	1
LP	0.7147	5
HP	0.7744	4

a Northeast Plain (NP), Inner Mongolia Plateau (IMP), Northwest Desert Oasis (NO), Loess Plateau (LP) and Huanghuaihai Plain (HP).

b is the weighted grey correlative degree, and is the grey relational coefficients matrix of indices, is the weight of the evaluated indices.

### 3.6 Comprehensive evaluation of alfalfa hay contamination in different regions

To evaluate the safety of alfalfa hay samples in the five regions, mycotoxin, bacterial, and heavy metal contents were analysed. As shown in Table 3, there were no significant differences among the five regions regarding AFB1, ZEN, TBC, TMC, As, Cd, Pb, and Cu contents (*p* > 0.05). Alfalfa hay samples in the IMP region had the highest DON content, whereas those in the LP region had the lowest DON content (*p* < 0.05). Cd content was higher in the LP region than in the NP region (*p* > 0.05). The Cr content in the NP region was significantly higher than that in the other four regions (*p* < 0.05). Samples from the LP and HP regions had higher Zn content than those from the NO region (*p* < 0.05). AFB1, DON, and ZEN contents detected in all five regions were within the safety range. All sampled alfalfa hay had more TBC and TMC contents than the limits of feed standards, and all heavy metal contents in the Table fall within the safety range, except for Cr, which exceeded the safety limit (27).

**Table 3:** Safety components of alfalfa hay samples.

Item^1^	Regions^2^				
	NP	IMP	NO	LP	HP
AFB1 (μg·kg^−1^)	4.04 ± 0.12	4.21 ± 0.55	3.76 ± 0.47	4.45 ± 0.39	4.36 ± 0.65
DON (mg·kg^−1^)	2.86 ± 0.49^bc^	4.39 ± 0.46^a^	3.56 ± 0.92^ab^	2.10 ± 0.27^c^	3.8 ± 1.32^ab^
ZEN (μg·kg^−1^)	65.41 ± 22.01	51.91 ± 11.89	50.04 ± 18.32	56.21 ± 12.08	64.37 ± 24.04
TBC (logCFU·g^−1^)	6.45 ± 1.78	6.51 ± 1.24	8.04 ± 1.01	6.85 ± 2.18	6.63 ± 1.03
TMC (log CFU·g^−1^)	4.78 ± 0.92	4.85 ± 1.31	5.01 ± 0.72	5.16 ± 0.99	5.16 ± 0.68
As (μg·kg^−1^)	59.53 ± 11.92	124.76 ± 63.62	125.55 ± 87.85	76.00 ± 53.78	120.49 ± 89.52
Cd (μg·kg^−1^)	131.95 ± 85.28	134.57 ± 122.29	133.94 ± 203.20	277.10 ± 204.72	94.70 ± 79.15
Pb (mg·kg^−1^)	3.12 ± 0.21	3.11 ± 0.60	3.42 ± 0.43	3.05 ± 0.38	3.01 ± 0.64
Cr (mg·kg^−1^)	74.11 ± 80.26^a^	14.16 ± 10.08^b^	9.52 ± 8.95^b^	5.44 ± 4.15^b^	24.81 ± 21.77^b^
Cu (mg·kg^−1^)	6.86 ± 1.99	7.84 ± 1.75	8.25 ± 1.43	8.18 ± 1.89	8.60 ± 1.91
Zn (mg·kg^−1^)	19.21 ± 5.34^ab^	21.10 ± 4.94^ab^	15.21 ± 3.37^b^	25.58 ± 2.81^a^	25.38 ± 5.19^a^

1 AFB1, aflatoxin B1; DON, deoxynivalenol; ZEA, zearalenone; TBC, total bacteria count; TMC, total mold count; As, arsenic; Cd, Cadmium; Pb, lead; Cr, chromium; Cu, copper; Zn, zinc.

2 Northeast Plain (NP), Inner Mongolia Plateau (IMP), Northwest Desert Oasis (NO), Loess Plateau (LP) and Huanghuaihai Plain (HP).a,b,c Means ± SD within rows with different superscript letters differ (*p* < 0.05).

According to the Nemerow contamination index method (Equations 4, 5), the contamination results of alfalfa hay samples showed that the NP and HP regions had heavily polluted alfalfa hay, followed by IMP (moderate contamination), NO (slight contamination), and LP (alert) regions (Table 4). Simultaneously, data on alfalfa hay contamination were analysed (Table 5). The detection rates of mycotoxins, microorganisms, and heavy metals in all samples were 100%, among which AFB1, ZEN, As, Cd, Pb, Cu, and Zn contents did not exceed the standard. The over-standard rate of DON was 6%, and its average content was still within the limit; however, the over-standard rates of TBC, TMC, and Cr were 66.7, 75.8, and 87.9%, respectively, and the average content exceeded the limit value, especially of the heavy metal Cr, which far exceeded the limit value of 5 mg kg^−1^.

**Table 4:** Evaluation results of Nemerow comprehensive contamination index method.

Item^a^	P_i_ max^b^	P_i_ ave^b^	I^b^	Classification	Contamination grade
NP	14.822	1.6528	10.5457	V	Heavy
IMP	2.8320	0.5970	2.0465	IV	Moderate
NO	1.9040	0.5212	1.3959	III	Slight
LP	1.1217	0.4233	0.8478	II	Alert
HP	4.9620	0.7895	3.5528	V	Heavy

a Northeast Plain (NP), Inner Mongolia Plateau (IMP), Northwest Desert Oasis (NO), Loess Plateau (LP) and Huanghuaihai Plain (HP).

b where is the over-limit ratio of safety component of alfalfa hay, is the species of safety component, is the tested single safety component concentration of alfalfa hay, and is the limited standard of safety component of alfalfa hay; is the quality of various comprehensive contamination indexes, represents the maximum value of one safety component, is the average value.

**Table 5:** Contamination situation of alfalfa hay.

Item^a^	Detection rate	Over-limit rate	Maximum value	Mean value
AFB1 (μg·kg^−1^)	100%	0%	6.16	4.19 ± 0.57
ZEN (μg·kg^−1^)	100%	0%	109.94	58.88 ± 20.17
DON (mg·kg^−1^)	100%	6%	6170.00	3607.88 ± 1149.63
TBC (logCFU·g^−1^)	100%	66.7%	9.49	6.86 ± 1.32
TMC (logCFU·g^−1^)	100%	75.8%	6.30	5.03 ± 0.84
As (μg·kg^−1^)	100%	0%	299.19	110.75 ± 76.47
Cd (μg·kg^−1^)	100%	0%	537.88	130.18 ± 131.21
Pb (mg·kg^−1^)	100%	0%	3.89	3.12 ± 0.53
Cr (mg·kg^−1^)	100%	87.9%	189.91	24.31 ± 35.07
Cu (mg·kg^−1^)	100%	0%	13.23	8.15 ± 1.79
Zn (mg·kg^−1^)	100%	0%	33.46	22.02 ± 5.97

a AFB1, aflatoxin B1; DON, deoxynivalenol; ZEA, zearalenone; TBC, total bacteria count; TMC, total mold count; As, arsenic; Cd, Cadmium; Pb, lead; Cr, chromium; Cu, copper; Zn, zinc.

### 3.7 Cluster analysis of alfalfa hay in different regions

**Figure 6:**
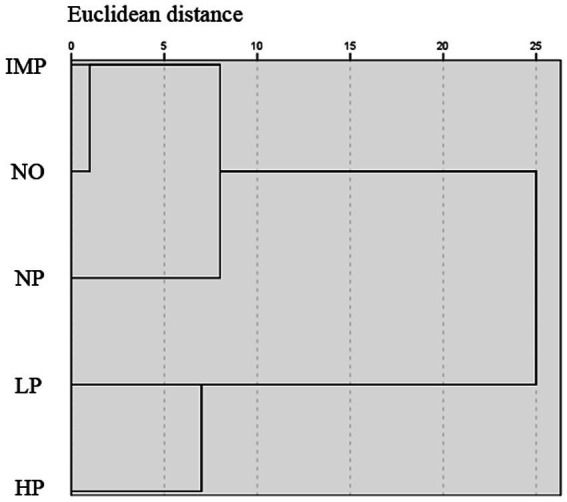
Cluster analysis on alfalfa hay nutritional value and safety index among the five areas. The five areas include the Northeast Plain (NP), Inner Mongolia Plateau (IMP), Northwest Desert Oasis (NO), Loess Plateau (LP), and Huanghuaihai Plain (HP). In the cluster diagram, the horizontal axis represents Euclidean distance, and the vertical axis represents 5 natural regions.

R-type clustering was used to reduce the dimensions of the nutritional components and safety indices of alfalfa hay. After selecting the main variables, Q-cluster was used to analyse the five regions and construct a distance spectrum clustering diagram (28). In the cluster diagram, the horizontal axis represented the Euclidean distance, and the vertical axis represented the five natural regions. The nearest region has a higher degree of similarity, which is clustered into a class, while the region far away has a low degree of similarity and therefore is clustered into a separate class. The regions were classified on the premise that the Euclidean distance of merging is less than 10. Cluster analysis of alfalfa hay nutrients and safety index showed three clusters among five sampling regions (Figure 6), which included Cluster I (IMP and NO), Cluster II (NP), and Cluster III (LP and HP). The results of the cluster analysis were consistent with the results of the correlation degree of alfalfa hay nutritional value.

## 4 Discussion

### 4.1 Nutritional value

In this study, CP, ADF, and Ash contents were not significantly different among the five sampling regions, whereas NDF, EE, and DM contents showed differences. NDF indicates palatability and digestibility potential. The lower the NDF, the better the alfalfa quality (29). Alfalfa hay sampled in the NO region had a higher CP content and lower NDF content, which is in accordance with the findings of a previous study (10). The arid moderate/warm temperate continental climate in this region contributes to a higher day/night temperature difference that facilitates DM accumulation. In addition, alfalfa hay in the HP and NP regions had higher EE contents than the NO and LP regions. Sun et al. (30) showed that higher EE content in alfalfa could be obtained with higher precipitation. The EE content was low in the NO region because of low precipitation. In contrast, although higher precipitation was recorded in the LP region, the EE content was still low owing to high evaporation in this region.

The mineral contents in alfalfa hay were affected by grassland and soil types, as well as the local climate (31, 32). In the current study, it was shown that the contents of Ca, P, K, Mg, and Zn in alfalfa samples were inevitably affected by sampling regions. As there are great differences in soil types and fertility in northern China, the enrichment ability and degree of mineral elements of alfalfa in different regions require further study. A previous study reported that Ca, P, and Ca: P ratios (less than 7) play important roles in the growth, development, and metabolism of livestock (33). In the LP region, the Ca, K, Mg, and P contents in alfalfa were lower than those in the other three regions. Care should be taken when using alfalfa hay from the LP region to guarantee the requirements for Ca and P intake.

With the increasing interest of consumers in the FA composition and content in meat and dairy products, an increasing number of studies have focused on FA, especially PUFA, *α*-linolenic acid, and linoleic acid. Linoleic acid and α-linolenic acid belong to n-6 PUFA and n-3 PUFA, respectively, which cannot be synthesised *de novo* in mammals and can only be taken from food. Compared with other forages, the content of polyunsaturated fatty acids in alfalfa is higher, especially linoleic acid and α-linolenic acid, and the fatty acid composition is more stable (34). Therefore, it is crucial to analyse the contents and composition of alfalfa FA for the optimisation of FA content in livestock products (35). PUFA was shown to lower cholesterol and increase immune system (36), whereas SFA has a cholesterol-raising effect. Our study showed that alfalfa hay in the NP region had the highest SFA and lowest PUFA levels, while the converse was observed in alfalfa hay in the IMP region (highest PUFA and lowest SFA levels). This might be due to the higher light intensity at higher altitudes (1000–1700 m in IMP vs. 120–300 m in NP), which contributes to the accumulation of PUFA (37, 38). This might also be because PUFA reached its peak in the full blossom stage (39).

There are 18 types of AAs in alfalfa stems and leaves. The diversity and content of AAs determine the quality of alfalfa (40). In our study, 17 types of AAs were identified. No significant differences were detected in the 17 AAs, essential AAs (EAAs), and total AAs (TAAs), which showed the stability of AA composition and content in all the five sampling regions.

In this study, the GRA method was employed to evaluate the nutritional value of alfalfa hay in different regions. GRA provided a comprehensive analysis of nutrients in alfalfa hay and ranked the nutrient values in different regions (24, 41). In our study, the nutrient values of alfalfa hay were ranked (highest to lowest) as NO > IMP > NP > HP > LP. In fact, the nutrient values of alfalfa hay are determined not only by intrinsic nutrient composition, but also by extrinsic factors such as insects and fertiliser use (42). The findings of this study could serve as a guide for the nutrient values of alfalfa hay in these sampling regions. As no other studies on nutrient values of alfalfa hay in different regions in China have been reported, it would be beneficial to integrate the nutrient states and geographical factors to provide guidelines for producing alfalfa hay with high nutrient content.

### 4.2 Alfalfa hay contamination

Safety has long been an important consideration in feed stocks. Chen et al. (43) studied mycotoxins in feed stocks in different seasons and found that mycotoxins could be identified in at least 70% of feed stocks. Previous studies have focused on mycotoxins in feed stocks such as corn and soybean meal, while few studies have investigated mycotoxins in alfalfa hay. Huang and Huang (44) studied mycotoxin contamination in feed stocks from 21 provinces and cities in China. They reported contamination by different mycotoxins in different regions and found that DON contamination was more severe than AFB1 and ZEN contamination. The differences in DON contamination caused by regional differences are consistent with the results of this study. However, all of the AFB1, DON, and ZEN contents in the sampled alfalfa hay were within the limits. Alfalfa cutting is often accompanied by rainfall, especially in the summer, making alfalfa hay vulnerable to rain, resulting in the production of mycotoxins during harvest, drying, and storage. For example, HP is located in humid or semi-humid warm temperate monsoon climate zones, with its second and third cutting periods falling between rainy and hot summer periods. To reduce potential mycotoxin contamination, it would be better to harvest the second cutting at the early flowering stage and extend the third cutting to the full blossom stage to make silage rather than alfalfa hay.

Microbes can also reduce the quality of alfalfa hay. Zhang (45) investigated the microbial changes in alfalfa bales with different moisture and storage temperatures and showed that TBC and TMC were positively correlated with temperature and moisture; the best group for microbial reproduction was at 35% moisture and 35°C. In the present study, both TBC and TMC contents were within the safety threshold. However, all samples in this study were collected in the hottest summer period with suitable temperature and humidity for microbial reproduction. Therefore, attention should be paid to store alfalfa hay during this season.

According to statistics by Li et al. (46), the polluted cultivated land area in China was 1.0 × 10^7^ hm^2^, among which industrial wastewater, chemical fertilisers and pesticides, and animal manure were the main sources of soil contamination. Research has shown that heavy metals preferentially accumulate in the roots and main-stem leaves of alfalfa (47). In recent years, heavy metals in alfalfa have been increasing owing to soil contamination and evolutionary adaptation under heavy metal stress (48). In our study, six heavy metals (As, Cd, Pb, Cr, Cu, and Zn) were detected in five sampling regions, of which Cr content in all areas exceeded the standard, especially in the NP region, which has been an industrial region for quite a long time (49). Heavy metal contamination is a long-term irreversible contamination that promotes the absorption and accumulation of heavy metals in alfalfa hay. However, there are significant variety differences in the absorption and accumulation of heavy metals in alfalfa (50). Prioritizing the selection of varieties with strong resistance to heavy metals is one of the effective ways to reduce heavy metal residues in alfalfa hay.

Individual areas in China have carried out comparative studies on the nutritional value of different alfalfa varieties (30, 51, 52). However, there has been no evaluation of the nutritional value of alfalfa bales in different regions, and no systematic evaluation of the degree of alfalfa bale contamination in different regions. Therefore, the Nemerow comprehensive contamination index method was used to determine the contamination degree of alfalfa bales, which was helpful in correctly judging the safety of alfalfa bales in these areas and attracting growing attention. In the present study, apart from the LP region, there was a trend of light to heavy contamination from the west to east region. Based on the analysis of alfalfa hay contamination data, the detection rate of mycotoxins, microorganisms, and heavy metals in all samples was 100%, and the TBC, TMC, and Cr contents in the five areas and DON content in the HP area all exceeded the standard and require increasing attention.

According to the cluster analysis, three clusters were formed among the five sampling regions. Alfalfa hay in the IMP and NO regions, which belonged to the first cluster, had a higher evaluation of nutritional value and safety performance. In the NP region, which belonged to the second cluster, alfalfa hay had high nutritional value but serious heavy metal contamination. Alfalfa hay in the HP and LP regions belonged to the third cluster, mainly because of its low nutritional value. At present, there are no reports on the nutritional value and contaminant evaluation of alfalfa hay in China. For commercial production, it is necessary to understand the actual nutritional value and contaminants of alfalfa hay in different regions for implementing more accurate and effective improvement measures in actual production and providing a scientific basis to produce high-quality alfalfa hay. Due to the limitation of funds, this study did not detect the soil conditions of the five major planting areas, and the impact of soil contamination on alfalfa hay can only be referred to by relevant literature. Obviously, the problem of soil contamination cannot be fixed within a short time, and increasing attention should be paid to its harmful effects on alfalfa quality and animal health.

## 5 Conclusion

In the current study, the comprehensive performance of nutritional value and safety of alfalfa hay was better in the IMP and NO regions. However, the phenomenon of alfalfa hay contamination is still widespread, and both internal and external factors contribute to the contamination. Therefore, for businesses growing alfalfa, varieties with strong resistance to heavy metals should be preferably selected based on the local contamination status. Additives can be used during the storage of alfalfa hay to prevent the proliferation of bacteria and moulds. Alternatively, alfalfa silage can be used instead of alfalfa hay during the rainy season. Detox or biodegradable reagents should be used to reduce the intake of mycotoxin pollutants in livestock. This study establishes the nutrient composition and harmful component profiles of alfalfa hay from 44 enterprises in five major planting areas in China for the first time, which provides a valuable reference for the safety and sustainable utilisation of alfalfa in food and livestock feed and provides insights to government administrators and enterprise managers for establishing alfalfa quality monitoring systems.

## Data Availability

The original contributions presented in the study are included in the article/Supplementary material, further inquiries can be directed to the corresponding authors.
