# Supplementary material for: Comprehensive evaluation of the nutritional value and contaminants of alfalfa (Medicago sativa L.) in China
Source: Front Nutr. 2025 Feb 10;12:1539462. doi: 10.3389/fnut.2025.1539462 (PMC11847697; doi:10.3389/fnut.2025.1539462)
Supplement: Supplementary file 1 [file Table_1.docx]

Supplementary Material

# Supplementary Tables

**Table S1** Limited standard of safety ingredients in plant feed raw materials

| **Item^1^** | **Enforcement basis^2^** | **Limit** |
| --- | --- | --- |
| AFB1 (μg·kg ^-1^) | GB 13078-2017 | 30 |
| ZEN (μg·kg ^-1^) | GB 13078-2017 | 1000 |
| [DON](javascript:;) (μg·kg ^-1^) | GB 13078-2017 | 5000 |
| TBC (logCFU·g^-1^) | GB 13078-2017 | 6.30 |
| TMC (logCFU·g^-1^) | GB 13078-2017 | 4.60 |
| As (ug·kg ^-1^) | GB 13078-2017 | 4000 |
| Cd (ug·kg ^-1^) | GB 13078-2017 | 1000 |
| Pb (mg·kg^-1^) | GB 13078-2017 | 30 |
| Cr (mg·kg^-1^) | GB 13078-2017 | 5 |
| Cu (mg·kg^-1^) | GB 26419-2010 | 35 |
| Zn (mg·kg^-1^) | NY 929-2005 | 250 |

^1^ AFB1 = aflatoxin B1; DON = deoxynivalenol; ZEA = zearalenone; TBC = total bacteria count; TMC = total mold count; As = arsenic; Cd = Cadmium; Pb = lead; Cr = chromium; Cu = copper; Zn = zinc.

^2^ GB 13078-2017, Hygienical standard for feeds, National standards of the P.R.C; GB 26419-2010, Limited contents of copper in feeds, National standards of the P.R.C; NY 929-2005, Limited contents of zinc in feeds, Industry standard of the Ministry of agriculture of the P.R.C.

**Table S2** Classification [standard](file:///F:\AppData\Local\youdao\DictBeta\Application\7.0.0.1203\resultui\dict\result.html) of Nemerow comprehensive pollution index

| **Pollution index** | **Classification** | **Pollution grade** |
| --- | --- | --- |
| $I$ ≤ 0.7 | Ⅰ | Safety |
| 0.7 <$I$ ≤ 1 | Ⅱ | Warning line |
| 1 <$I$≤ 2 | Ⅲ | Light pollution |
| 2 <$I$≤ 3 | Ⅳ | Medium [pollution](javascript:;) |
| $I$>3 | Ⅴ | [Heavy](javascript:;) [pollution](javascript:;) |

**Table S3** Dimensionless data processing

| **Item^1^** | **Dimensionless data^2^** | | | | | |
| --- | --- | --- | --- | --- | --- | --- |
|  | **X_1_** | **X_2_** | **X_3_** | **X_4_** | **X_5_** | **X_0_** |
| CP | 0.9617 | 0.9805 | 0.9733 | 0.9361 | 0.9821 | 0.9733 |
| NDF | 0.9952 | 0.9892 | 0.9657 | 0.9217 | 0.9973 | 0.9657 |
| ADF | 0.9639 | 0.9794 | 0.9506 | 0.9649 | 0.9872 | 0.9506 |
| Ca | 0.9776 | 0.9618 | 0.9776 | 0.7099 | 0.9632 | 0.9632 |
| P | 1.0000 | 0.9565 | 0.8148 | 0.7273 | 1.0000 | 0.8148 |
| EAA | 0.9966 | 0.9165 | 0.9668 | 0.9656 | 0.9777 | 0.9165 |
| α-linolenic | 0.8108 | 0.7644 | 0.9526 | 0.9857 | 0.9475 | 0.7644 |

^1^ CP = crude protein; NDF = neutral detergent fiber; ADF = acid detergent fiber; EAA = essential amino acid.

^2^ X_1_ = nutritional index of NP region/mean value of each nutrient index in NP region; X_2_ = nutritional index of IMP region/mean value of each nutrient index in IMP region; X_3_ = nutritional index of NO region/mean value of each nutrient index in NO region; X_4_ = nutritional index of LP region/mean value of each nutrient index in LP region; X_5_ = nutritional index of HP region/mean value of each nutrient index in HP region; X0 = mean value of each nutrient index in each region/optimal values of each nutrition index in each region.

**Table S4** Absolute differences between *X_0_* and *X_1_*

| **Item^1^** | **Absolute differences^2^** | | | | |  |
| --- | --- | --- | --- | --- | --- | --- |
|  | ***△_1_*** | ***△_2_*** | ***△_3_*** | ***△_4_*** | ***△_5_*** | |
| CP | 0.0116 | 0.0072 | 0.0000 | 0.0372 | 0.0088 | |
| NDF | 0.0295 | 0.0235 | 0.0000 | 0.0440 | 0.0316 | |
| ADF | 0.0133 | 0.0288 | 0.0000 | 0.0143 | 0.0366 | |
| Ca | 0.0144 | 0.0014 | 0.0144 | 0.2533 | 0.0000 | |
| P | 0.1852 | 0.1417 | 0.0000 | 0.0875 | 0.1852 | |
| EAA | 0.0801 | 0.0000 | 0.0503 | 0.0491 | 0.0612 | |
| α-linolenic | 0.0464 | 0.0000 | 0.1882 | 0.2213 | 0.1831 | |

^1^ CP = crude protein; NDF = neutral detergent fiber; ADF = acid detergent fiber; EAA = essential amino acid.

^2^ Absolute differences of each nutrient index in each region, $\Delta_{i}(k)=|X_{0}(k)-X_{i}(k)|$ (i = 1, 2, 3, 4, 5; k = 1, 2, 3, 4, 5)

**Table S5** Correlative coefficients of each nutrition composition

| **Item^1^** | **Correlative coefficients^2^** | | | | |  |
| --- | --- | --- | --- | --- | --- | --- |
|  | **ε_1_** | **ε_2_** | **ε_3_** | **ε_4_** | **ε_5_** | |
| CP | 0.9161 | 0.9460 | 1.0000 | 0.7729 | 0.9353 | |
| NDF | 0.8109 | 0.8434 | 1.0000 | 0.7420 | 0.8005 | |
| ADF | 0.9051 | 0.8149 | 1.0000 | 0.8988 | 0.7757 | |
| Ca | 0.8978 | 0.9893 | 0.8978 | 0.3334 | 1.0000 | |
| P | 0.4061 | 0.4719 | 1.0000 | 0.5913 | 0.4061 | |
| EAA | 0.6127 | 1.0000 | 0.7158 | 0.7205 | 0.6743 | |
| α-linolenic | 0.7318 | 1.0000 | 0.4022 | 0.3640 | 0.4089 | |

^1^ CP = crude protein; NDF = neutral detergent fiber; ADF = acid detergent fiber; EAA = essential amino acid.

^2^Correlative coefficients of each nutrition composition in five regions,

$\varepsilon_{i}\left( k \right)=\frac{\min\left( i \right)\min\left( k \right)\left| X_{0}\left( k \right)-X_{1}\left( k \right) \right|+\rho\max\left( i \right)\max\left( k \right)\left| X_{0}\left( k \right)-X_{1}\left( k \right) \right|}{\Delta_{i}\left( k \right)+\rho\max\left( i \right)\max\left( k \right)\left| X_{0}\left( k \right)-X_{i}\left( k \right) \right|}$, $\rho$= 0.5, $min\left( i \right)min\left( k \right)\left| X_{0}(k)-X_{i}(k) \right|$ = 0.0000, $max(i)max(k)|X_{0}(k)-X_{i}(k)|$ = 0.2533.
